# Pre- and post-contrast T1 mapping for detection of myocardial fibrosis in severe aortic stenosis before and after aortic valve replacement

**DOI:** 10.1186/1532-429X-16-S1-M7

**Published:** 2014-01-16

**Authors:** Emmanuelle Vermes, Aurelia Ros, Julien Pucheux, Anne Delhommais, Daniel Alison, Olivier Genee

**Affiliations:** 1 Cardiac Imaging, University Hospital of Trousseau, Tours, France

## Background

Severe aortic stenosis (AS) leads to diffuse fibrosis in the myocardium, which could cause poor clinical outcome. Cardiovascular Magnetic Resonance (CMR) with recently T1 mapping is a promising technique to identify diffuse myocardial fibrosis. The Objective of the present study was to assess myocardial fibrosis by CMR using T1 mapping in patients with surgical AS and evaluate the evolution after aortic valve replacement (AVR).

## Methods

A prospective CMR pre- and post-contrast T1 mapping study of 15 patients with severe AS was conducted before and six months after AVR and in 15 controls. CMR at 1.5 T, including T1 mapping using a shortened modified Look-Locker inversion recovery sequence, was carried out. Segmental and global T1 values have been measured before and 15 minutes after the administration of 0.2 mmol/kg of Gadoteric acid. T1 values have been compared before and six months after AVR.

## Results

Mean non-contrast global T1 values were significantly higher in the AS group before AVR (970 ± 28 ms) than in controls (943 ± 25 ms) (p = 0.005). Six months after surgery, mean T1 values in the anteroseptal segment significantly decreased (from 967 ± 58 ms to 932 ± 42 ms) (p = 0.03). There were no significant differences on global T1 values after surgery (970 ± 29 ms before, 968 ± 32 ms after; p = 0.42). T1 post-contrast global values were not significantly different before and after surgery.

## Conclusions

In surgical aortic stenosis, pre-contrast T1 mapping suggests the presence of diffuse myocardial fibrosis. Six months after surgery, pre-contrast segmented based T1 mapping suggests that fibrosis could be partially reversible. Post-contrast T1 mapping does not provide any additional information.

## Funding

No funding.

